# Spontaneous Facial Mimicry Is Enhanced by the Goal of Inferring Emotional States: Evidence for Moderation of “Automatic” Mimicry by Higher Cognitive Processes

**DOI:** 10.1371/journal.pone.0153128

**Published:** 2016-04-07

**Authors:** Aiko Murata, Hisamichi Saito, Joanna Schug, Kenji Ogawa, Tatsuya Kameda

**Affiliations:** 1 Department of Behavioral Science, Hokkaido University, Sapporo, Hokkaido, Japan; 2 Japan Society for the Promotion of Science, Tokyo, Japan; 3 Department of Psychology, The College of William & Mary, Williamsburg, Virginia, United States of America; 4 Department of Psychology, Hokkaido University, Sapporo, Hokkaido, Japan; 5 Department of Social Psychology, The University of Tokyo, Tokyo, Japan; University of Pisa, ITALY

## Abstract

A number of studies have shown that individuals often spontaneously mimic the facial expressions of others, a tendency known as facial mimicry. This tendency has generally been considered a reflex-like “automatic” response, but several recent studies have shown that the degree of mimicry may be moderated by contextual information. However, the cognitive and motivational factors underlying the contextual moderation of facial mimicry require further empirical investigation. In this study, we present evidence that the degree to which participants spontaneously mimic a target’s facial expressions depends on whether participants are motivated to infer the target’s emotional state. In the first study we show that facial mimicry, assessed by facial electromyography, occurs more frequently when participants are specifically instructed to infer a target’s emotional state than when given no instruction. In the second study, we replicate this effect using the Facial Action Coding System to show that participants are more likely to mimic facial expressions of emotion when they are asked to infer the target’s emotional state, rather than make inferences about a physical trait unrelated to emotion. These results provide convergent evidence that the explicit goal of understanding a target’s emotional state affects the degree of facial mimicry shown by the perceiver, suggesting moderation of reflex-like motor activities by higher cognitive processes.

## Introduction

In human societies there is a continual need to coordinate and cooperate with other non-kin individuals in a wide range of social settings. To coordinate effectively with others while minimizing the potential risk of exploitation, individuals must accurately understand the intentions and emotions of others [1]. While in some instances intentional effort is required to infer the thoughts and feelings of others, in many cases it seems that people can understand each other’s feelings quickly and effortlessly [2]. Spontaneous facial mimicry is considered a key process in the quick and effortless understanding of others’ feelings, as well as in the fostering of bonding with partners [3], along with other forms of physiological mimicry such as synchronization of heartbeat [4] and pupil diameter [5].

Spontaneous facial mimicry could be induced by motor resonance mechanisms grounded in automatic perception-action coupling in the sensorimotor regions [6]. The discovery of “mirror neurons” in monkeys, which are activated during both action observation and production [7], as well as human brain regions having similar properties [8], have given neurophysiological support to direct action-perception matching. In line with these neural findings, a number of studies suggest that facial mimicry may be an automatic, fast, reflex-like mechanism beyond intentional control. For instance, Dimberg and colleagues [9] and Bailey and Henry [10] showed that participants exhibited facial mimicry even when facial stimuli were presented subliminally, and that participants’ muscular movements started within 500ms after stimulus onset [9]. These results suggest that the process occurs largely outside of conscious control. The occurrence of rapid mimicry has also been recently demonstrated in many other non-human mammals, including apes [11, 12], monkeys [13], and dogs [14]. It is also observable quite early in development, as early as the neonatal stage in humans [15] as well as in chimpanzees [16], suggesting that facial mimicry occurs automatically as a reflex-like reaction.

Recently, however, several studies on humans have demonstrated that facial mimicry is affected by socio-ecological factors, including the social relationship between the sender and the receiver, group membership, and so on (see [17] for a recent comprehensive review). For example, Bourgeois and Hess showed that people tended to mimic the facial expressions of in-group members more frequently than those of out-group members [18]. Hofman and colleagues also demonstrated that facial mimicry was affected by the target’s reputation for fairness: compared to a baseline, participants exhibited greater facial mimicry when angry faces of unfair opponents were shown, while mimicry decreased when angry faces of fair opponents were shown [19].

These findings indicate that spontaneous facial mimicry may be moderated by the observers’ tasks or relational goals in social contexts [3, 17]. For example, correctly identifying the emotional states of in-group members, with whom we exchange key resources regularly, is presumably more important than understanding the emotional state of out-group members, with whom we are likely to have little or no contact. Likewise, when interacting with individuals known to have engaged in unfair or dishonest behavior, the necessity for vigilance against potential exploitation and aggression is enhanced. Thus it may be the case that mimicking the negative emotions of unfair targets may prepare us to guard against a potentially exploitative interaction, while matching the anger of fair individuals may be damaging to a potentially beneficial interaction. Although this interpretation is highly speculative, such differential incentive structures related to specific socio-ecological contexts [20, 21] may have contributed to the differential mimicry levels of negative emotions between the fair and unfair individuals observed by Hofman and colleagues [19].

Here we investigate the hypothesis that spontaneous facial mimicry may be moderated by the observer’s goal of understanding a target’s emotional state. Although it has been demonstrated that blocking observers’ facial muscle activity impairs their ability to recognize a target’s expressed emotions [22], there have been few studies that directly address the adjustment of mimicry level in response to the specific goal of understanding another’s emotional state. The only exception, as far as we know, is a study by Cannon, Hayes and Tipper [23], in which participants were explicitly asked to judge either the emotional states of targets (i.e., anger and happiness) or the color of tinted facial photographs. Results showed that participants exhibited greater facial mimicry when they engaged in the emotion-judgment task than in the color-judgment task. Here we aim to examine the robustness of this intriguing finding by extending the target facial stimuli to various emotional expressions beyond anger and happiness. As in Cannon and colleagues [23], we measure participants’ facial muscle activity while they view video clips of targets displaying facial expressions, but we use six target expressions rather then two: happiness, sadness, anger, disgust, fear, and surprise. In Study 1, we use electromyography (EMG) to examine the degree of facial mimicry exhibited by participants when they are explicitly instructed to infer the target’s emotional state, compared to when they receive no such instruction. In Study 2, we introduce another condition in which participants are instructed to infer non-emotional traits of the target (e.g., age, gender, physical attribute, or ethnicity) before the video presentation, and their facial muscle activity is assessed using the Facial Action Coding System (FACS), a less invasive procedure than EMG. We predict that the extent of participants’ facial mimicry will be greater when participants have the specific goal of inferring the targets’ emotional states, compared to when they receive no such instruction, or when they have another goal unrelated to emotional inference.

## Study 1

### Materials and Methods

#### Ethics statements

Study 1 and Study 2 were both approved by the Institutional Review Board of the Center for Experimental Research in Social Sciences at Hokkaido University. Written informed consent was obtained from all participants before beginning the task.

#### Participants

Fifty-two Japanese student volunteers (26 females and 26 males; mean age: 19.2 ± 1.1 years) at Hokkaido University in Sapporo participated in this experiment and received 1,000 yen (approximately US$10 at the time) as compensation for their participation. Electromyographic (EMG) data from two participants were excluded due to equipment failure, yielding a total of 50 participants (25 females and 25 males) for analysis.

#### Stimuli

Twenty-four morphing video clips of emotional facial expressions were presented to each participant. Morphing video clips were created using facial photos of eight Japanese targets (4 females and 4 males with ages ranging from mid-20s to mid-30s) from the ATR Facial Expression Image Database DB99 (ATR-Promotions, Inc.). For each of six types of emotional expressions (happiness, sadness, anger, disgust, fear, and surprise), participants saw 4 video clips of two female and two male targets (see S1 Table for details about how the eight target persons were assigned to the six types of emotional expressions).

#### Facial EMG

**Fig 1:**
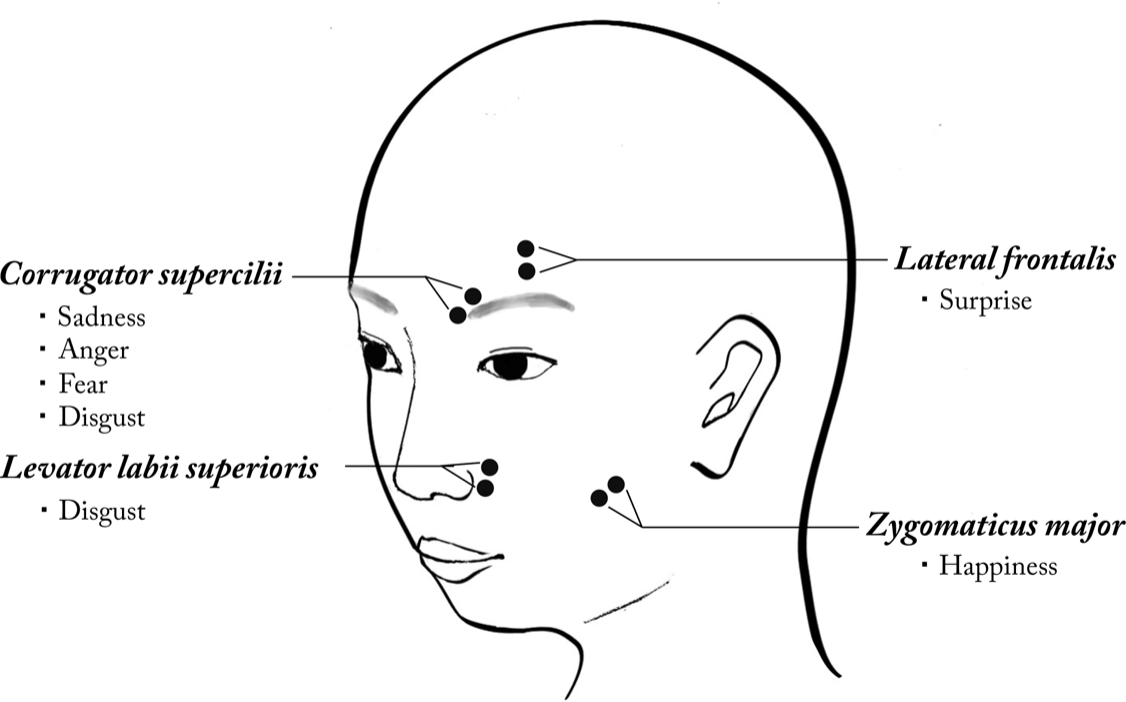
EMG electrode placement and emotional expression measurement in Study 1. Activity of the *Zygomaticus major* was measured to assess smiling (related to happiness); activity of the *Corrugator supercilii* was measured to assess frowning (related to anger, disgust, sadness, and fear); activity of the *Levator labii Superioris* was measured to assess upper lip raising (related to disgust); and activity of the *Lateral frontalis* was measured to assess eyebrow raising (related to surprise).

EMG recordings were performed while participants viewed the stimuli. Facial EMG was measured on the left side of the face, which has been shown to exhibit a higher mimicry rate as compared to the right side [24]. As shown in Fig 1, electrodes were placed according to the standard procedure [25]. The activities of four muscles of interest (see Fig 1) were measured using Ag / AgCl miniature surface electrodes (EL254S, BIOPAC Systems Inc.) with electrolyte gel (Elefix, Nihon Kohden). The skin was cleansed with disinfectant alcohol and pumice gel (Skin Pure, Nihon Kohden). An AcqKnowledge System with a band pass filter was used to exclude EMG signals outside the relevant range of 1-500Hz. The EMG signals were sampled at 200Hz, integrated with 12.5Hz, then rectified and averaged over 100ms intervals.

#### Procedure

Participants were randomly assigned either to the Passive condition or the Emotion-Inference condition. After arriving in the laboratory, each participant was taken to a soundproof room and seated in front of a computer. The participant’s face was video-recorded using a camera mounted on the left side of the computer monitor (Qcam Orbit AF, Logitech) throughout the tasks, in order to determine whether facial or body movements irrelevant to facial expression (e.g., yawning, blinking) occurred.

**Fig 2:**
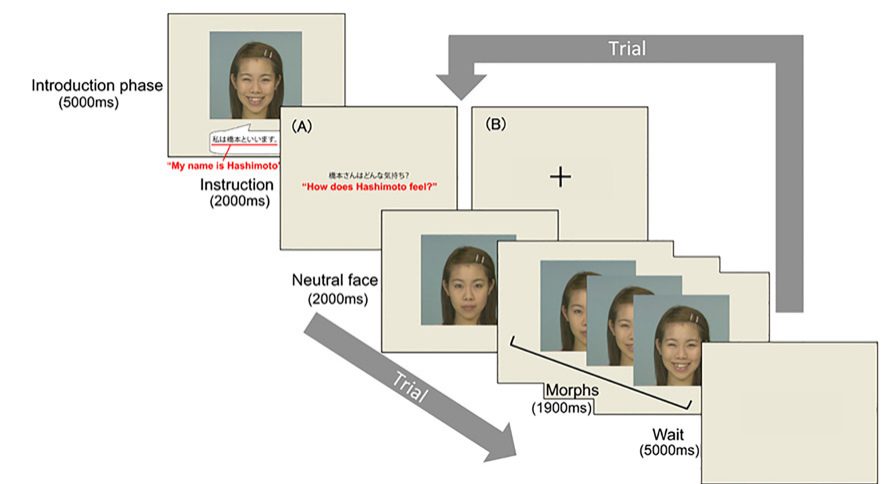
Task flow in Study 1. At the beginning of each block, an introduction-picture was presented, followed by 2–4 trials. (A) In the Emotion-Inference condition, the instruction “How does XXX (e.g., Hashimoto) feel?” was presented in Japanese. (B) In the Passive (control) condition, a fixation cross was presented. Reprinted from the ATR Facial Expression Image Database DB99 under a CC BY license, with permission from ATR-Promotions Inc., original copyright (2006).

The experimental task consisted of eight blocks, each containing between two and four trials. In each block, various emotional expressions (one per trial) of the same target (see S1 Table) were presented sequentially (see Fig 2). At the beginning of each block, participants were shown an introductory image for 5000ms, which consisted of a photo of the target’s smiling face and a self-introduction text in Japanese for the target (e.g., “My name is Hashimoto”), to familiarize participants with the target’s face before viewing the video clips in the following trials. The order of blocks and trials within each block were counterbalanced across participants.

In the Emotion-Inference condition (see A in Fig 2), each trial started with a display of instructions in Japanese, which lasted for 2000ms, explicitly asking participants to infer the emotion felt by the target (e.g., “How does Hashimoto feel?”). In the Passive condition, a fixation cross was presented for 2000ms instead of the instructions (see B in Fig 2). Next, a video clip began with the first frame showing a neutral facial expression lasting for 2000ms, followed by a morph from a neutral to an emotional expression, during which the target’s facial expression changed gradually from neutral to full over 1000ms and stayed at the full expression for the remaining 900ms. After this, a blank screen was shown between trials for 5000ms. Thus, except for the instructions, the procedure and stimuli used were identical across the two conditions.

#### Data treatment and analysis

In each trial, the EMG data collected during the 4000ms after the start of the video clip (3900ms for the duration of the clip plus an extra 100ms at the beginning of the waiting period) were z-transformed within each participant and each muscle to permit comparison of activities between the four muscles (see Fig 1). For each trial, the response window was the 1900ms interval during which the face changed from a neutral expression to a full expression. If the video of the participant’s face showed irrelevant facial activity during the response window (i.e., blinking, yawning or turning their eyes away), the associated EMG data were excluded. Because EMG wave amplitudes sometimes exhibit abnormal values due to equipment error [25], if the mean z-score of a participant’s muscle activity was deviant (i.e., more than 3 SD away) from the mean of the muscle activities averaged across all participants, the participant’s data from that muscle site were treated as missing values (though preliminary analyses including these data produced statistically the same conclusions). Each participant’s facial muscular response per trial was calculated by averaging muscle activity during the 1900ms interval after the morphing onset. If a participant mimicked the target’s facial expression, the activities of the facial muscles corresponding to the stimulus expression should be selectively enhanced. Therefore, in the following analysis, we compared the activities of “targeted muscles” used in the movements of each of the emotional-expression stimuli (see Fig 1), with the activities of “non-targeted muscles” during the response window, and we refer to this difference as the muscle “type.”

We used generalized linear mixed effects models (GLMM) to analyze EMG activity for each muscle type. Condition, muscle type (targeted vs. non-targeted) and emotion were entered as fixed effects. Because we had repeated measures from the same participants, and trials were nested within each participant, participants and trials were both treated as random effects in the models. Because facial muscular responses are measured as continuous values ranging from negative to positive, GLMMs were modeled with Gaussian distributions and fitted using the GLIMMIX procedure in SAS statistical software version 9.4 (SAS Institute, Cary, NC).

In the GLMM analysis, the models of all possible combinations of fixed factors and interactions were fitted and compared in terms of the degree of fit according to the Akaike information criterion ([26]; see S2 Table for details about the model selection). If occurrences of facial mimicry are moderated by the conditions as predicted, the best-fit model should include the interaction effect of condition (Passive vs. Emotion-Inference) and muscle type (targeted vs. non-targeted).

### Results

**Fig 3:**
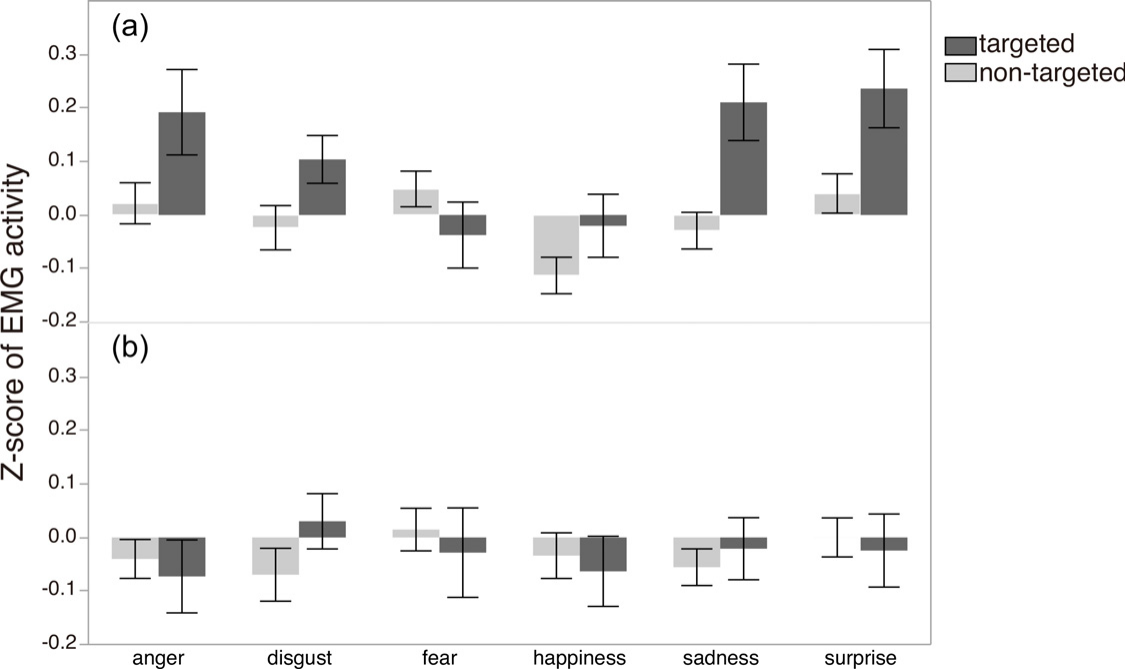
**Z scores of EMG activity by emotion for each muscle type (targeted or non-targeted) in (a) the Emotion-Inference condition (N = 26) and (b) the Passive condition (N = 24)**. Error bars represent standard error of the mean.

Fig 3 shows z-scores of EMG activity for each muscle type (targeted/non-targeted) as a function of the six emotions and two conditions. Consistent with our hypothesis, targeted muscle activity was generally higher than non-targeted in the Emotion-Inference condition, while no such effects were evident in the Passive condition. The GLMM analysis supported this observation: the best-fit model (see S2 Table and S1 Fig for details about the model selection), contained the expected condition x muscle type interaction effect (*F*_3, 4580_ = 8.71, *p* < .0001; see S3 Table for parameter coefficients of the selected model). The effect of emotion was also significant (*F*_5, 4580_ = 2.62, *p* = .023), indicating that the magnitudes of muscular responses were different across the six emotions.

**Fig 4:**
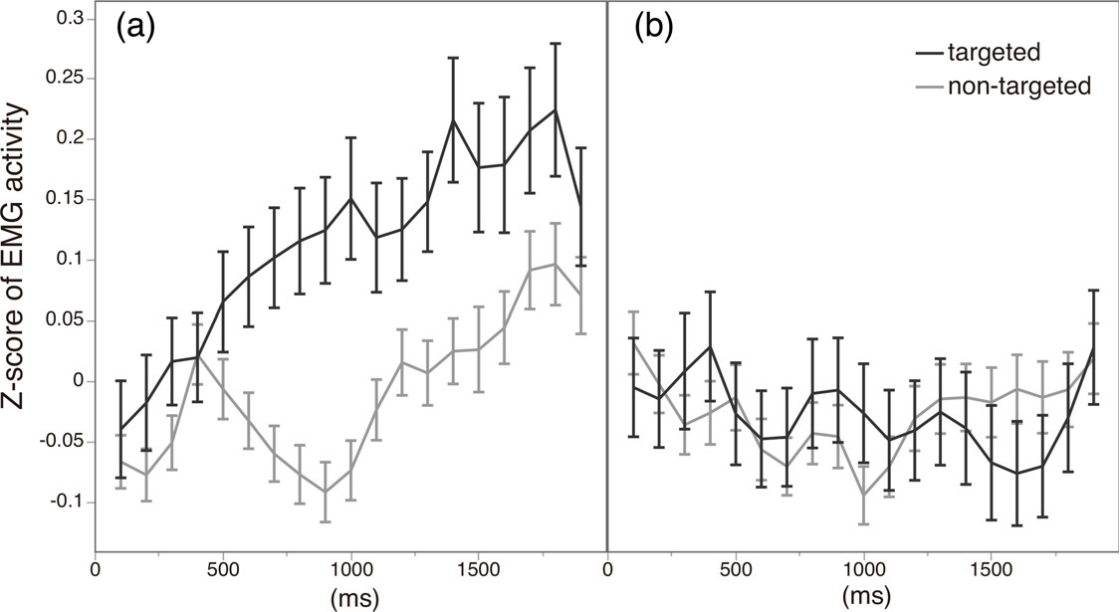
**Time course of EMG activity of targeted and non-targeted muscles from morphing onset in (a) the Emotion-Inference condition and (b) the Passive condition.** The horizontal axis shows time elapsed from morphing onset (in milliseconds), and the vertical axis shows z-score of EMG amplitude for each muscle type. Error bars represent standard error of the mean at each time point.

To investigate in more detail how the emotion-inference goal may moderate facial mimicry, we examined changes in the EMG signals over time. Fig 4 shows the time course of EMG signals of the two types of muscle (targeted vs. non-targeted) under the two conditions. In the Emotion-Inference condition, differences in EMG activities between the two types of muscle started to emerge at about 500ms after the onset of morphs. In contrast, the activity of the targeted muscles in the Passive condition remained indistinguishable from the activity of the non-targeted muscles. This indicates that, when participants were instructed to infer the target’s emotion, facial mimicry measured as muscular EMG activity emerged rapidly, immediately after the stimulus (morphing) onset.

## Study 2

We conducted Study 2 to further test the validity of the moderation effect of goal-setting on facial mimicry as observed in Study 1, with some methodological modifications. The first modification in Study 2 was the use of the Facial Action Coding System (FACS) to assess the facial expressions displayed by participants in response to stimuli. While the EMG measurements used in Study 1 are known to be a sensitive method of detecting both visible and invisible muscular activity, EMG signals may also include noise from body movements irrelevant to emotional expression (e.g., eye blinks, yawns, etc.). Indeed, given the potential for noise in the data, the number of trials per emotion used in Study 1 was relatively low compared to typical EMG emotion research [9, 18, 19], although we have addressed this problem statistically by using a multi-level model that fully captured the nested structure of the data set. Because FACS is a method to specifically code visible facial muscular movements, it allows us to accurately evaluate facial activity with less noise than EMG, as well as to examine whether the goal-dependent moderation of mimicry is evoked in externally visible facial reactions.

Furthermore, we could not completely dismiss the possibility that the effects of the goal-related instructions in Study 1 were merely caused by differences in participants’ levels of concentration between passively viewing morphs and being asked to actively attend to the target’s face, rather than the motivation to infer the target’s emotion. In other words, it is possible that the participants in the Passive condition who received no specific instruction may simply have been less engaged in the task compared with participants in the Emotion-Inference condition. To address this potential problem, the second modification in Study 2 was to ask participants to respond to questions about the facial morph stimuli in both the experimental and control conditions. For the control, we asked participants to reply to questions about external traits that were irrelevant to emotional inference (i.e., age, gender, body shape, and ethnicity) but required the same level of concentration, to ensure that they were given similar motivation to engage in the task.

### Materials and Methods

#### Participants

Fifty-five Japanese student volunteers (26 females and 29 males; mean age: 19.27 ± 2.18 years) at Hokkaido University in Sapporo participated in this experiment and received 1,000 yen (approximately US$10 at the time) as compensation for their participation. Participants were randomly assigned to one of the two conditions.

#### Procedure

The procedure was almost identical to that of Study 1 with a few methodological modifications. First, as discussed above, participants’ facial expressions were recorded using a camera mounted above the computer display (Webcam Pro 9000, Logitech) for coding using FACS, without any electrodes attached to their faces. Second, participants in the control condition were asked to answer questions about the target’s non-emotional traits (age, gender, body shape, and ethnicity: see S4 Table). One of these four trait-questions was pre-assigned randomly to the 24 video clips, and was displayed before the clip started. Participants in the experimental condition were asked to answer questions about the target’s emotional states, and the facial stimuli and the time sequence used in Study 2 were kept identical to those of Study 1.

#### Facial Action Coding System (FACS)

The facial expressions of participants were recorded over the course of the experimental tasks and coded using the Facial Action Coding System Manual [27]. FACS is an anatomy-based system for comprehensively describing visible facial muscular movements in terms of Action Units (AUs). We coded four AUs corresponding to the targeted muscles in Study 1 (see S2 Fig): AU 4 for *Corrugator supercilii* (brow lowering, associated with anger, sadness, disgust, and fear), AU 12 for *Zygomaticus major* (lip corner raising, associated with happiness), AU10 for *Levator labii superioris* (upper lip raising, associated with disgust), AU2 for *Lateral frontalis* (brow raising, associated with surprise).

#### Data acquisition and analysis

Two scorers who were trained in FACS coding but blind to the conditions made binary judgments about whether or not each AU of the participant’s face was active within the 1900ms interval following the morphing onset, during which the target’s face changed from a neutral expression to a full expression. Scores for which there were disagreements between the two scorers were re-coded by both scorers independently, and inter-scorer reliability for the coding was sufficiently high for all AUs (Cronbach’s alpha: AU2, 0.95; AU4, 0.94; AU10, 0.82; AU12, 0.97). The following analyses used coding results that were consistent across the two scorers. If a participant covered a part of his/her face with a hand, or the eyebrow was covered by hair, the scores of the corresponding AUs were treated as missing values.

As in Study 1, we used GLMM to analyze AU activation rate by type. Condition, AU type (targeted vs. non-targeted), and emotion were entered as fixed effects, and participants were treated as random effects. As a measure of the degree of facial mimicry, we used the rate (out of 4 trials per emotion) of each AU movement in response to each emotional expression. Thus, GLMMs were modeled using logit link functions with binomial distributions, and fitted using the GLIMMIX procedure in SAS. The models of all possible combinations of fixed factors and interactions were fitted and compared in terms of the degree of fit by the Akaike information criterion (AIC) as in Study 1.

### Results

To assess differences in task difficulty and its potential effects on participants’ attention levels between the two conditions, we compared the accuracy of participants’ judgments in each task. As shown in S3 Fig, there was no significant difference in mean accuracy between the two conditions (*M* = 0.801, *SE* = 0.018 in the Emotion-Inference condition and *M* = 0.803, *SE* = 0.018 in the Trait-Judgment condition). This result confirms that task demand was equivalent between the two conditions.

**Fig 5:**
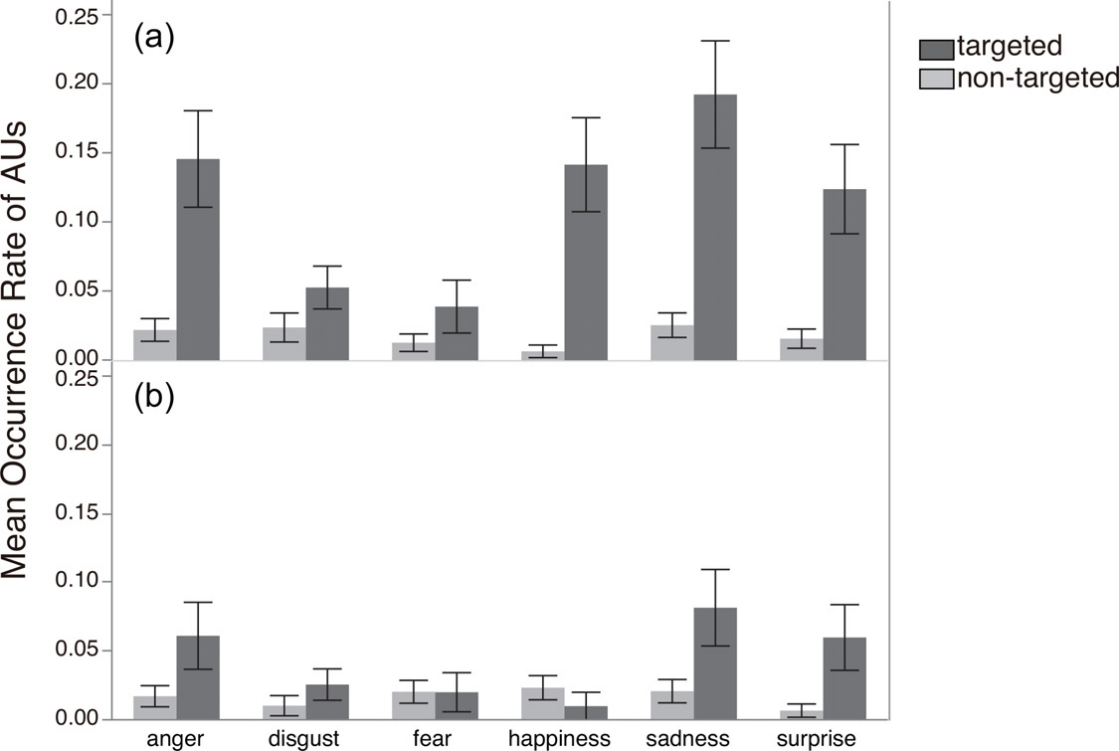
**Mean occurrence rates of each AU type (targeted and non-targeted) when participants were (a) inferring emotional states (N = 28) or (b) judging external traits (N = 27) of the targets.** Error bars represent standard error of the mean.

Fig 5 displays the mean activation rate of each AU as a function of the six emotional expressions and the two conditions. As shown in the figure, activation of the targeted AUs was generally higher than activation of the non-targeted AUs in the Emotional-Inference condition. In contrast, such effects were generally less evident in the Trait-Judgment condition.

The GLMM analysis supported this observation. The best-fit model (see S5 Table for details about the model selection) contained the condition x AU type interaction effect (*F*_3, 52_ = 36.49, *p* < .0001) and the effect of emotion (*F*_5, 266_ = 4.95, *p* = .0002; see S6 Table for parameter coefficients of the selected model). Notice that the best-fit model in Study 2 was identical to that of Study 1, thus clearly replicating its results using FACS coding (rather than EMG) and the Trait-Judgment condition (rather than the Passive condition) as a control for methodological modifications.

## Discussion

The results from these two experiments consistently showed that the degree of facial mimicry increased when participants were given the explicit goal of inferring targets’ emotional states. In Study 1, emotion-specific facial muscle activity measured by EMG (corresponding to the facial muscle activity in the stimuli) was enhanced when participants were explicitly asked to infer the target’s emotion, relative to when they were assigned no specific goal. As in previous studies [9, 19], facial mimicry measured using EMG activity started to emerge quite early after the stimulus onset (about 500ms after the morphing started). The effect of the instructions also emerged soon after stimulus onset: as shown in Fig 4, differences in facial EMG activity patterns between the two conditions became evident about 500-1000ms after the morphing started. Taken together, these results suggest that the explicit goal of inferring another’s emotional state promotes participants’ readiness to attend to emotional expression in advance (whether consciously or unconsciously), thus facilitating the subsequent facial mimicry process.

Study 2 addressed several potential methodological issues in Study 1. It could be argued that the participants who were given no explicit goal in the Passive condition of Study 1 may simply have been less focused on the task and stimuli compared to participants in the Emotion-Inference condition. In Study 2, we addressed this concern by introducing a new control condition in which participants were explicitly instructed to make a judgment regarding the external traits of the target (i.e., age, gender, body shape, or ethnicity) rather than infer their emotional state. We verified that task difficulty, which might have affected participants’ attention to the facial stimuli, was equivalent between the two conditions (S3 Fig). We also used the Facial Action Coding System (FACS), instead of EMG, in Study 2. Because FACS is a method of coding only visible facial muscular movements, it allows us to examine whether the goal-related moderation of mimicry was evoked in externally recognizable facial reactions. The results from Study 2 corroborated the results from Study 1, supporting our hypotheses that facial mimicry is facilitated not by general attention to the target but by the specific goal of inferring the emotional state of the target, and that such enhanced mimicry is also visually recognizable. It should also be noted that the enhanced mimicry effect was observed generally across the six emotions (anger, disgust, fear, happiness, sadness, surprise), substantially extending the previous findings on anger and happiness [23].

Taken together, the overall results suggest that human facial mimicry, which has often been considered a reflex-like phenomenon (similar to the mirror system [8] shared with non-human animals and other forms of physiological mimicry such as synchronization of heartbeat [4] and pupil diameter [5, 28]), can be moderated by the specific situational goal of inferring another’s emotional state. In a similar vein, Hess and Fischer have argued that emotional mimicry (of which facial mimicry is a major component) is “related to the understanding of an emotion in context and is involved in regulating one’s relation with the other person, rather than being the synchronization of meaningless individual muscle actions” ([3]: pp.144-146). In other words, as compared to simple motor mimicry, emotional mimicry seems to be much more socially nuanced and affected by various socio-ecological factors [17].

Still, there are limitations and lingering questions in this research. First, in both studies reported here, some muscles were designated as targeted muscles across several emotions (e.g., *Corrugator supercilii* and AU4 for sadness, anger, fear and disgust; see Figs 1 and S2). Thus, even if *Corrugator supercilii* became active when participants observed an angry target face, it could be argued that this may actually have been a fearful response rather than mimicry of anger, which cautions us against a strong interpretation of the results as mimicry [3]. Because the activation pattern we had hypothesized for mimicry was observed robustly for the other targeted muscles as well, which correspond uniquely to each of the specific emotions (i.e., *Lateral frontalis* and AU2 for surprise, *Levator labii superioris* and AU10 for disgust, and *Zygomaticus major* and AU12 for happiness), we think that our overall conclusion is adequate. Nevertheless, in future research, it would be desirable to address discrete facial expression more precisely by analyzing combinatorial patterns of multiple muscular activities [3] with advanced image-processing technology [29].

Second, in the passive condition of Study 1, where participants saw only a fixation cross before the morphing started, no mimicry effect was observed (i.e., no difference in EMG activity between the targeted and non-targeted muscles; see Fig 3). This result may be seen as a replication failure of the basic facial-mimicry phenomenon [9, 10]. Although we don’t have a direct explanation for the absence of mimicry here, it seems worth mentioning that previous studies using Japanese participants have generally tended to show low occurrence of facial mimicry in the laboratory [30, 31]. For example, in a study by Sato and Yoshikawa, no facial mimicry (measured in differential muscle activity in AU4 and AU12 between anger and happiness) was observed among participants in the “static” condition, who simply observed targets’ faces on a computer screen for 1520ms; muscle movement was also generally low, occurring in only 2–8% of all trials [31]. It seems useful to note that, compared with historically heterogeneous cultures such as the US and Canada, individuals in historically homogeneous cultures such as Japan and China tend to avoid both explicitly showing emotion in public [32] and staring directly at others’ faces [33–35]. Thus the absence of facial mimicry in the Passive condition of Study 1 may reflect such a cultural influence. Notice that facial mimicry was observed in the Trait-Judgment condition of Study 2, where focusing on the target’s face was required (and thus culturally justifiable) as a means to solve the task. Cultural moderation of facial mimicry as sketched here would seem to be an intriguing topic for future research.

Third, we did not observe a gender difference in the moderation effect of goal-setting on facial mimicry; female and male participants alike showed greater mimicry when they were explicitly instructed to infer targets’ emotions than otherwise. The only gender-related effect we observed was a marginal effect in Study 1, showing that females tended to move muscles more to all facial stimuli (not selectively as implied by facial mimicry) than males (see the note accompanying S2 Table). This result is consistent with several previous studies showing that females exhibited greater facial muscle reactivity compared to males when exposed to facial expressions [36, 37]. Some studies have also suggested that males have more control over their emotional expressions than females [38–40]. On the other hand, gender effects on facial mimicry per se have been mixed in the previous literature (see [17] for a review). While in some studies females were reported to show greater facial mimicry than males [36], this gender effect was not replicated by other studies [41]. Given such mixed results, it would seem plausible that some socio-ecological factor may moderate the possible gender effect on facial mimicry, leaving this issue open for future research.

Fourth, in Study 2, there was no correlation between participants’ facial mimicry level and their accuracy in emotion recognition in the Emotion-Inference condition (*r* = -0.10, *p* = .59; S4 Fig). We speculate that this result might be due the high recognition accuracy (80% accuracy on average; see S3 Fig) for the facial stimuli that we used in this research. Given that previous studies which also used easily-recognizable, prototypical facial displays have reported the absence of this correlation [42, 43], we conjecture that using ambiguous or non-prototypical facial stimuli that elicit lower mean accuracy may be necessary to detect the possible mimicry-accuracy relation [3].

Lastly, we conjecture that the cognitive moderation of facial mimicry in relation to specific task goals, as demonstrated here, may help us solve different and nuanced situational demands efficiently. Developmental psychologists have shown that infants exhibit facial mimicry almost automatically even when there is no explicit goal of inferring a target’s emotional state (e.g., [15]), while the mimicry shown by adult participants in our studies was affected by their specific task goals [3, 17]. We speculate that such differences between infants and adults may indicate regulatory processes coming into play, which change the initial reflex-like mimicry into a more elaborate response, as our brains mature during development and socialization. In line with this speculation, neuroscientists have identified two separate neural circuits that help us understand the minds of others: the “experience sharing” network, which simulates a target’s internal states as our own bodily feelings, including the anterior insula (AI), anterior cingulate cortex (ACC), and inferior frontal gyrus (IFG) (see [44] for review); and the “mentalizing” network, which infers a target’s mental states, including the medial prefrontal cortex (MPFC), the temporo-parietal junction (TPJ) and the medial parietal cortex (see [45] for review). Although the potential interaction of these two circuits is considered to be an important requirement for “higher-order” human empathy [46], little is known about the mechanisms of interaction between the two systems (though see [47]). The goal-dependent moderation of facial mimicry observed in the current studies may reflect such interactive processes, in which top-down, cognitive goal-setting for understanding the target’s emotional state meets bottom-up, physical mimicry. Future research employing neuroimaging techniques and physiological measurements with an experimental protocol similar to the one we have developed here may be useful to illuminate such an interplay with greater precision at multiple levels.

## Supporting Information

S1 Dataset Dataset of Study 1. (XLSX)

S2 Dataset Dataset of Study 2. (XLSX)

S1 Fig **Mean Z scores of EMG activity for each muscle type (targeted or non-targeted) by gender, collapsed over emotions, in (a) the Emotion-Inference condition (N = 26) and (b) the Passive condition (N = 24).** Error bars represent standard error of the mean.
(EPS)

S2 Fig Examples of facial stimuli used in the two studies. Correspondence between the targeted muscles in Study 1 and the targeted AUs in Study 2 are shown. The electrode placements for each of the targeted EMG measurements in Study 1 are shown on the right side of each picture, in red. The targeted Action Units in Study 2 are shown on the left side of each picture, in blue. AU4: *Corrugator supercilii* (CS; brow lowering, targeted AU for anger, disgust, fear, and sadness), AU2: *Lateral frontalis* (LF; brow raising, targeted AU for surprise), AU10: *Levator labii Superioris* (LS; upper-lip raising, targeted AU for disgust), and AU12: *Zygomaticus major* (ZM; lip-corner raising, targeted AU for happiness). Reprinted from the ATR Facial Expression Image Database DB99 under a CC BY license, with permission from ATR-Promotions Inc., original copyright (2006).
(EPS)

S3 Fig Mean recognition accuracy in the Emotion-Inference condition (N = 28) and the Trait-Judgment condition (N = 27) in Study 2. Error bars represent standard error of the mean. There was no difference in recognition accuracy between the conditions, *t*(53) = 0.09, *p* = 0.93.
(EPS)

S4 Fig Correlation between individual recognition accuracy and degree of facial mimicry (mean activity-rate of the targeted AUs) in the Emotion-Inference condition (N = 28) in Study 2. *r* = -.10, *p* = .59.
(EPS)

S1 Table Assignment of the eight target persons in the stimuli set to each of the six emotional expressions. (PDF)

S2 Table Model selection by Generalized Linear Mixed Model analysis of EMG data using GLIMMIX procedure with Laplace approximation (*k*: number of parameters, log L*: Maximum log likelihood, AIC: Akaike information criterion, Rank_AIC_: rank order by AIC). (PDF)

S3 Table Parameter coefficients of the best-fit model in Study 1 (i.e., model 12 in S2 Table). Parameter coefficients related to the Condition x Muscle type interaction effect were calculated with the activities of non-targeted muscles in the Passive condition as a baseline. Parameter coefficients related to the effect of emotion were calculated with the activities related to surprised expressions as a baseline. Although AIC values were used for model selection (S2 Table), we also report marginal *F*-test statistics for the fixed factors of the selected model (model 12) to show the relative contribution of each effect.
(PDF)

S4 Table Instructions used in Study 2. In the Emotion-Inference condition, the question used was identical to the one in the Emotion-Inference condition of Study 1. In the Trait-Judgment condition, one of the four questions below was presented before the video clip was started. Response options for each question are shown on the right.
(PDF)

S5 Table Model selection by Generalized Linear Mixed Model analysis of FACS data using GLIMMIX procedure with Laplace approximation (*k*: number of parameters, log L*: Maximum log likelihood, AIC: Akaike information criterion, Rank_AIC_: rank order by AIC). (PDF)

S6 Table Parameter coefficients of the best-fit model in Study 2 (i.e., model 12 in S5 Table). Coefficients related to the Condition x AU Type interaction effect were calculated with the activities of non-targeted AUs in the Trait-Judgment condition as a baseline. Parameter coefficients related to the effect of emotion were calculated with the activities related to surprised expressions as a baseline. Although AIC values were used for model selection (S5 Table), we also report marginal *F*-test statistics for the fixed factors of the selected model (model 12) to show the relative contribution of each effect.
(PDF)
