# Supplementary material for: Spontaneous Facial Mimicry Is Enhanced by the Goal of Inferring Emotional States: Evidence for Moderation of “Automatic” Mimicry by Higher Cognitive Processes
Source: PLoS One. 2016 Apr 7;11(4):e0153128. doi: 10.1371/journal.pone.0153128 (PMC4824486; doi:10.1371/journal.pone.0153128)
Supplement: S1 Table — (PDF) [file pone.0153128.s007.pdf]

Target's gender	Target's ID in the ATR Facial Expression Image Database DB99	Emotional expressions
Female	f3	anger disgust fear surprise
	f10	happiness sadness surprise
	f13	anger disgust fear
	f16	happiness sadness
Male	m1	happiness sadness disgust
	m6	happiness disgust
	m9	sadness anger fear surprise
	m10	anger fear surprise
