# Supplementary material for: Spontaneous Facial Mimicry Is Enhanced by the Goal of Inferring Emotional States: Evidence for Moderation of “Automatic” Mimicry by Higher Cognitive Processes
Source: PLoS One. 2016 Apr 7;11(4):e0153128. doi: 10.1371/journal.pone.0153128 (PMC4824486; doi:10.1371/journal.pone.0153128)
Supplement: S2 Table — (PDF) [file pone.0153128.s008.pdf]

Model	Random effects	Fixed effects	k	Deviance $-2 \log L^*$	Residual deviance	AIC	Rank_{AIC}
model 1	Participants	Null	1	9078.16	62.71	9082.16	15
model 2	Trials	Condition	2	9070.33	54.88	9076.33	13
model 3	(Same for all	Muscle type	2	9067.51	52.06	9073.51	11
model 4	models)	Emotion	6	9064.99	49.54	9078.99	14
model 5		Condition Muscle type	3	9059.68	44.23	9067.68	7
model 6		Muscle type Emotion	7	9054.53	39.08	9070.53	8
model 7		Condition Emotion	7	9057.08	41.63	9073.08	10
model 8		Condition Muscle type Emotion	8	9046.61	31.16	9064.61	3
model 9		Condition x Muscle type	4	9052.03	36.58	9062.03	2
model 10		Emotion x Muscle type	12	9044.78	29.33	9070.78	9
model 11		Condition x Emotion	12	9048.97	33.52	9074.97	12
**model 12**		**Condition x Muscle type Emotion**	**9**	**9038.94**	**23.49**	**9058.94**	**1**
model 13		Condition Muscle type x Emotion	13	9036.84	21.39	9064.84	4
model 14		Condition x Emotion Muscle type	13	9038.47	23.02	9066.47	6
model 15		Full	24	9015.45	0	9065.45	5

Note. We have also conducted a GLMM analysis with participant's gender as an additional fixed effect. Because our hypothesis centered on the interaction effect between Condition and Muscle type, we tested whether there was a gender effect in relation to the Condition x Muscle type interaction. Two of the models that contained the effect of gender had slightly smaller AIC than model 12 (i.e., Model A: Condition x Muscle type, Emotion, Gender, AIC = 9057.90; Model B: Condition x Muscle type x Gender, Emotion, AIC = 9057.48). The contribution of the effect of gender in Model A was statistically marginal, $F_{1,4580} = 3.05$, $p = .081$, indicating that females tended to be more facially reactive to facial stimuli (but not selectively as implied by facial mimicry) than males. As seen in S1 Fig, the Condition x Muscle type x Gender effect in Model B was largely due to the negative mean EMG activity of the targeted muscles among males in the Passive condition. However, notice that the hypothesized Condition x Muscle type interaction is statistically robust for both males and females. Separate matched-pair T tests showed a significantly greater mean EMG activity of the targeted muscles than the non-targeted muscles in the Emotion-Inference condition (males: $t_{11} = 2.71$, $p = 0.02$; females: $t_{13} = 3.09$, $p = 0.01$) but not in the Passive condition (males: $t_{12} = -1.76$, $p = 0.11$; females: $t_{10} = 1.53$, $p=0.16$). Thus, for the sake of simplicity, we did not include those models that incorporated participant's gender in S2 Table.
