# Supplementary material for: Spontaneous Facial Mimicry Is Enhanced by the Goal of Inferring Emotional States: Evidence for Moderation of “Automatic” Mimicry by Higher Cognitive Processes
Source: PLoS One. 2016 Apr 7;11(4):e0153128. doi: 10.1371/journal.pone.0153128 (PMC4824486; doi:10.1371/journal.pone.0153128)
Supplement: S3 Table — Parameter coefficients related to the Condition x Muscle type interaction effect were calculated with the activities of non-targeted muscles in the Passive condition as a baseline. Parameter coefficients related to the effect of emotion were calculated with the activities related to surprised expressions as a baseline. Although AIC values were used for model selection (S2 Table), we also report marginal F-test statistics for the fixed factors of the selected model (model 12) to show the relative contribution of each effect. (PDF) [file pone.0153128.s009.pdf]

model 12

Parameters			Coefficient	*SE*	*95% Confidence Limits*		*df*	*F*	*p*
Intercept			0.02	0.03	-0.07	0.10	3		
Condition x Muscle Type	(Condition)	(Muscle Type)						8.71	<0.0001
	Emotion-Inference	Targeted	0.14	0.03	0.08	0.20	4580		
		Non-targeted	0.02	0.02	-0.02	0.06	4580		
	Passive	Targeted	0.01	0.03	-0.05	0.07	4580		
		Non-targeted	0.00						
Emotion								2.62	0.0225
	anger		-0.03	0.03	-0.10	0.03	4580		
	disgust		-0.05	0.03	-0.11	0.02	4580		
	fear		-0.03	0.03	-0.09	0.04	4580		
	happiness		-0.11	0.03	-0.18	-0.05	4580		
	sadness		-0.05	0.03	-0.11	0.01	4580		
	surprise		0.00						
