# Supplementary material for: Spontaneous Facial Mimicry Is Enhanced by the Goal of Inferring Emotional States: Evidence for Moderation of “Automatic” Mimicry by Higher Cognitive Processes
Source: PLoS One. 2016 Apr 7;11(4):e0153128. doi: 10.1371/journal.pone.0153128 (PMC4824486; doi:10.1371/journal.pone.0153128)
Supplement: S4 Table — In the Emotion-Inference condition, the question used was identical to the one in the Emotion-Inference condition of Study 1. In the Trait-Judgment condition, one of the four questions below was presented before the video clip was started. Response options for each question are shown on the right. (PDF) [file pone.0153128.s010.pdf]

Condition	Question (in Japanese)	Choices
Emotion-Inference	“How does this person feel? ”	happy / sad / angry / disgusted / fearful / surprised
Trait-Judgment	“Is this person female or male”	female / male / hard to tell
	“How old is this person?”	10's / 20's / 30's / 40's / 50's / 60's
	“What is this person’s ethnic background?”	European / Asian / African / Oceanian / Arabic
	“What body shape does this person have?"	tall and lean / average / stout / stocky
