# Supplementary material for: Spontaneous Facial Mimicry Is Enhanced by the Goal of Inferring Emotional States: Evidence for Moderation of “Automatic” Mimicry by Higher Cognitive Processes
Source: PLoS One. 2016 Apr 7;11(4):e0153128. doi: 10.1371/journal.pone.0153128 (PMC4824486; doi:10.1371/journal.pone.0153128)
Supplement: S5 Table — (PDF) [file pone.0153128.s011.pdf]

Model	Random effects	Fixed effects	k	Deviance $-2 \log L^*$	Residual deviance	AIC	$Rank_{AIC}$
model 1	Participants	Null	1	1018.78	158.45	1022.78	13
model 2	(Same for all	Condition	2	1015.13	154.8	1021.13	12
model 3	models)	AU Type	2	1034.53	174.2	1038.53	15
model 4		Emotion	6	1002.98	142.65	1016.98	11
model 5		Condition AU Type	3	918.69	58.36	926.69	9
model 6		AU Type Emotion	7	897.12	36.79	913.12	6
model 7		Condition Emotion	7	999.37	139.04	1015.37	10
model 8		Condition AU Type Emotion	8	893.42	33.09	911.42	4
model 9		Condition x AU Type	4	908.97	48.64	918.97	7
model 10		Emotion x AU Type	12	886.59	26.26	912.59	5
model 11		Condition x Emotion	12	997.14	136.81	1023.14	14
**model 12**		**Condition x AU Type Emotion**	**9**	**883.36**	**23.03**	**903.36**	**1**
model 13		Condition AU Type x Emotion	13	882.88	22.55	910.88	3
model 14		Condition x Emotion AU Type	13	891.07	30.74	919.07	8
model 15		Full	24	860.33	0	910.33	2

Note. We also conducted a GLMM analysis with participant's gender as an additional fixed effect. However, none of those models provided a better fit to the FACS data in terms of AIC than model 12 in the table. Thus, for the sake of simplicity, we did not include those models that incorporated participant's gender in S5 Table. We also conducted a GLMM analysis incorporating trials as random effects and treating FACS coding in each trial as a binary score. However, five of these models did not converge numerically to maximum likelihood estimates due to excessive zero frequencies. To solve this problem, we used the rate of AU movements in response to each emotional expression (out of 4 trials per emotion) as a measure of the degree of facial mimicry in the GLMM analysis.
