# Supplementary material for: Spontaneous Facial Mimicry Is Enhanced by the Goal of Inferring Emotional States: Evidence for Moderation of “Automatic” Mimicry by Higher Cognitive Processes
Source: PLoS One. 2016 Apr 7;11(4):e0153128. doi: 10.1371/journal.pone.0153128 (PMC4824486; doi:10.1371/journal.pone.0153128)
Supplement: S6 Table — Coefficients related to the Condition x AU Type interaction effect were calculated with the activities of non-targeted AUs in the Trait-Judgment condition as a baseline. Parameter coefficients related to the effect of emotion were calculated with the activities related to surprised expressions as a baseline. Although AIC values were used for model selection (S5 Table), we also report marginal F-test statistics for the fixed factors of the selected model (model 12) to show the relative contribution of each effect. (PDF) [file pone.0153128.s012.pdf]

model 12

Parameters			Coefficient	*SE*	*95% Confidence Limits*		*df*	*F*	*p*
Intercept			-4.87	0.41	-5.69	-4.05	54		
Condition x AU Type	(Condition)	(AU Type)						36.49	<.0001
	Emotion-Inference	Targeted	2.29	0.44	1.40	3.17	52		
		Non-targeted	0.10	0.46	-0.83	1.02	52		
	Trait-Judgment	Targeted	1.06	0.28	0.49	1.62	52		
		Non-targeted	0.00						
Emotion								4.95	.0002
	anger		0.27	0.28	-0.29	0.83	266		
	disgust		-0.63	0.31	-1.23	-0.02	266		
	fear		-0.55	0.34	-1.21	0.11	266		
	happiness		-0.08	0.30	-0.67	0.52	266		
	sadness		0.57	0.27	0.03	1.10	266		
	surprise		0.00						
